# Supplementary figures and images for: Multilevel regulation of the glass locus during Drosophila eye development
Source: PLoS Genet. 2019 Jul 12;15(7):e1008269. doi: 10.1371/journal.pgen.1008269 (PMC6655844; doi:10.1371/journal.pgen.1008269)

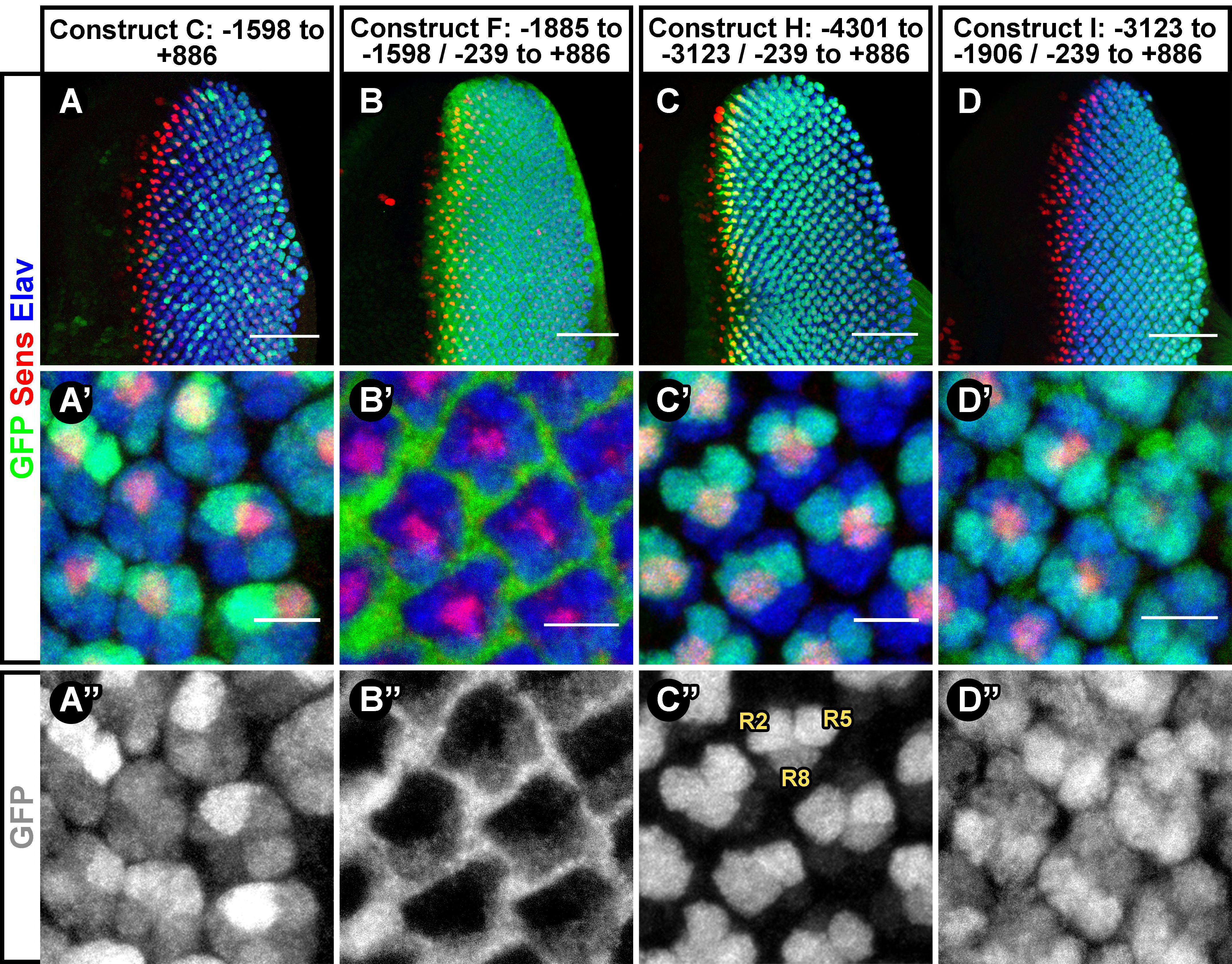

Supplement: S1 Fig — GFP (green), Senseless (Sens, red), and Elav (blue) expression in the eye region of larval imaginal discs of transgenic flies expressing construct C (-1598 to +886) (A), construct F (-1885 to -1598 / -239 to +886) (B), construct H (-4301 to -3123 / -239 to +886) (C), or construct I (-3123 to -1906 / -239 to +886) (D) (compare to Fig 2A for the individual constructs). Scale bar: 40 μm A’ to D’: magnification of areas in panels A to D. Scale bar: 5 μm. A” to D”: GFP channel alone. (TIF) [file pgen.1008269.s001.tif]

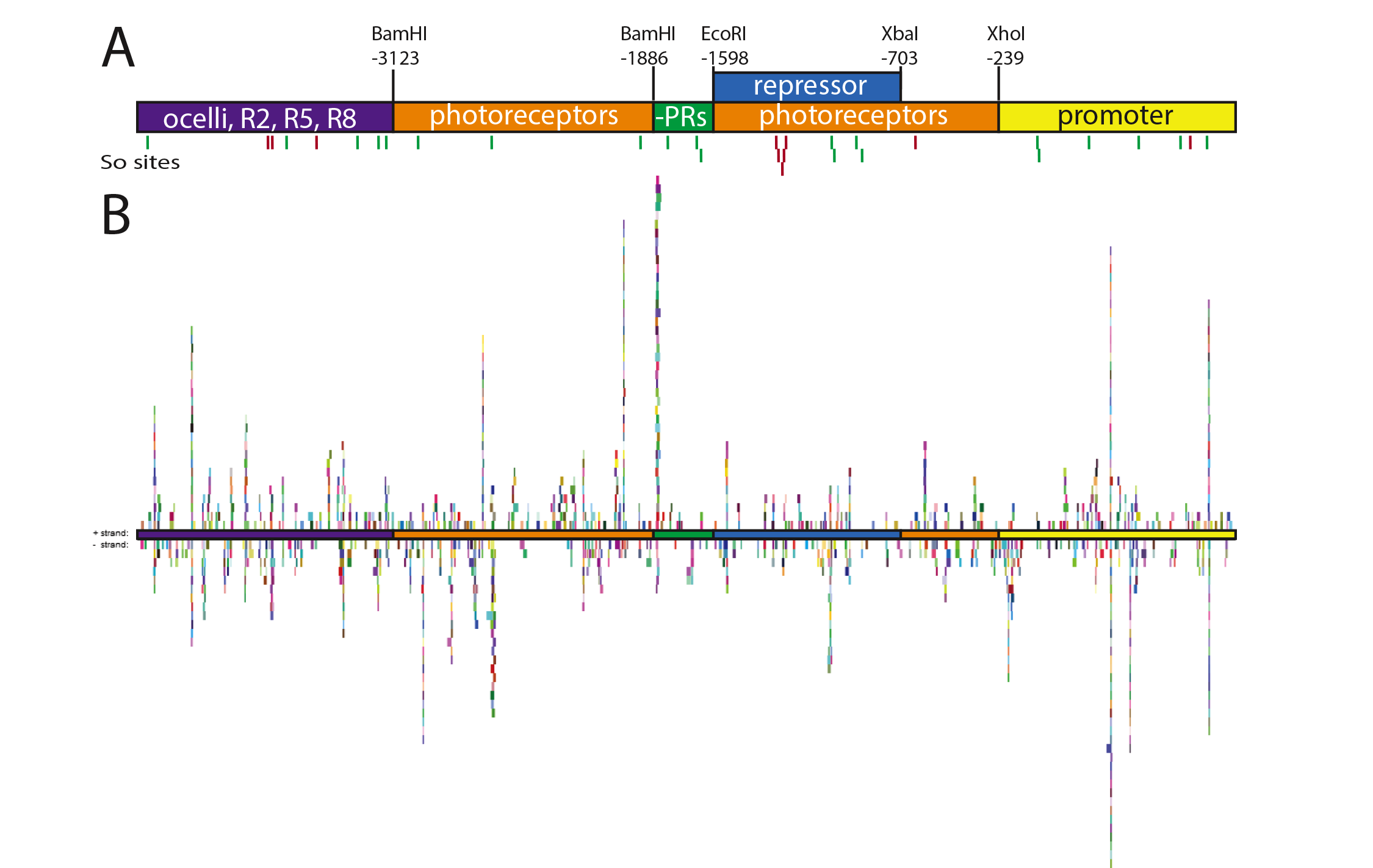

Supplement: S2 Fig — A: The 5.2 kb glass regulatory region contains different enhancer elements: an ocelli enhancer that also drives expression in a subset of photoreceptors (-4301 to -3123; purple), two photoreceptor enhancers (-3123 to -1886 and -1598 to -239; orange), a non-photoreceptor enhancer (-1886 to -1598, green), a repressor element (-1598 to -703; blue) and a promoter region (-239 to +886; yellow). There are 31 potential binding sites for the transcription factor Sine oculis within the entire 5.2 kb upstream genomic region; 10 sites with the consensus AGATAC (red bars) and 21 sites with the consensus YGATAY (green bars). B: in silico analysis of potential transcription factor binding sites. Binding sites are spread along the entire 5.2 kb region (coloured bars) with several clusters in the different enhancer fragments. (TIF) [file pgen.1008269.s002.tif]

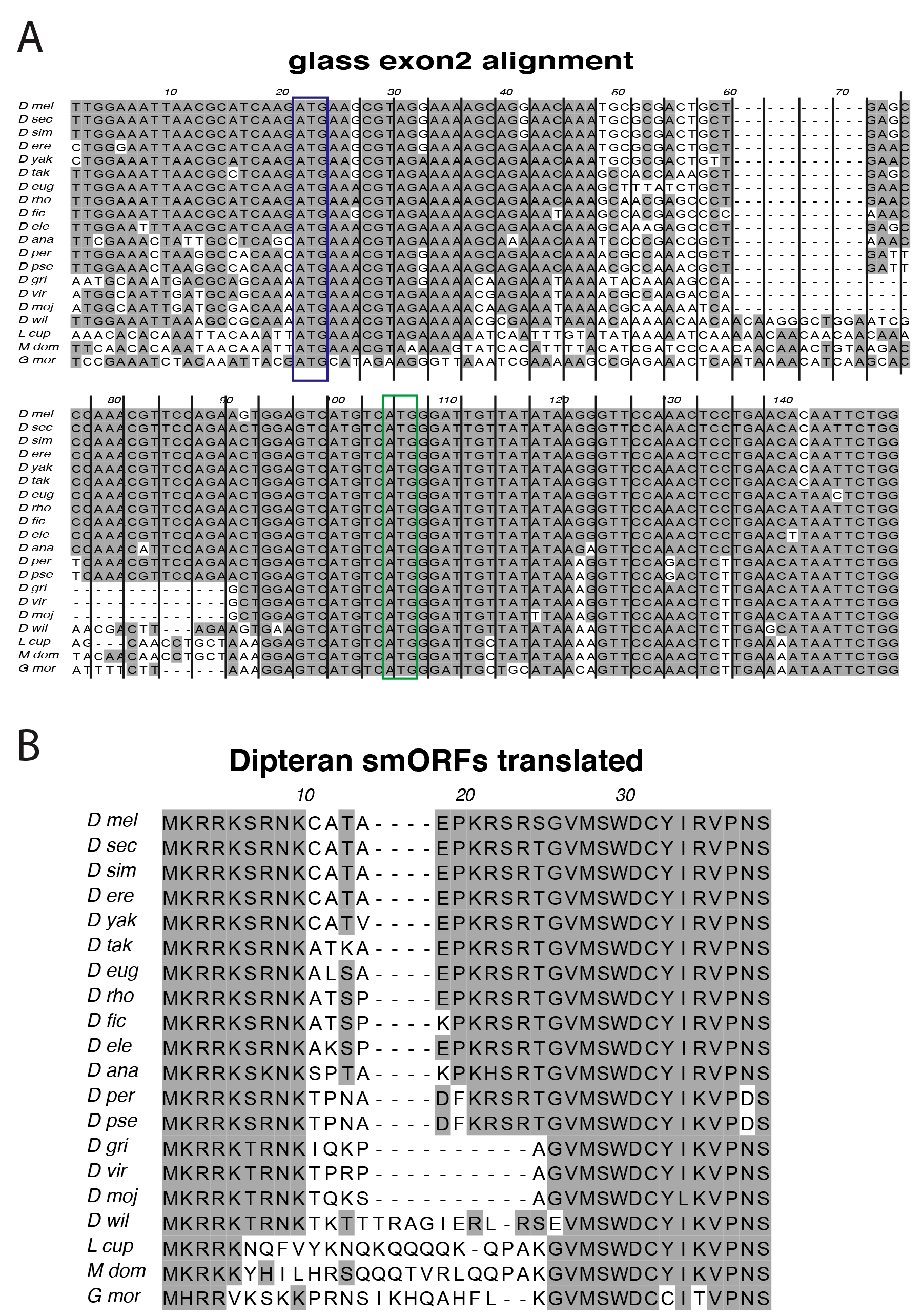

Supplement: S3 Fig — A: nucleotide sequence of glass exon 2 of different higher Diptera. The position of the glass start codon is outlined in green. The position of the upstream start codon is outlined in blue. The black vertical lines show the triplets following the upstream start codon that result in the amino acid sequences shown in B. B: Alignment of the amino acid sequences resulting from translation beginning at the start codon upstream of Glass of different higher Diptera. Drosophila melanogaster (D mel), Drosophila sechellia (D sec), Drosophila simulans (D sim), Drosophila erecta (D ere), Drosophila yakuba (D yak), Drosophila takahashii (D tak), Drosophila eugracilis (D eug), Drosophila rhopaloa (D rho), Drosophila ficusphila (D fic), Drosophila elegans (D ele), Drosophila ananassae (D ana), Drosophila persimilis (D per), Drosophila pseudoobscura (D pse), Drosophila grimshawi (D gri), Drosophila virilis (D vir), Drosophila mojavensis (D moj), Drosophila willistoni (D wil), Lucilia cuprina (L cup), Musca domestica (M dom), Glossina morsitans (G mor). (TIF) [file pgen.1008269.s003.tif]

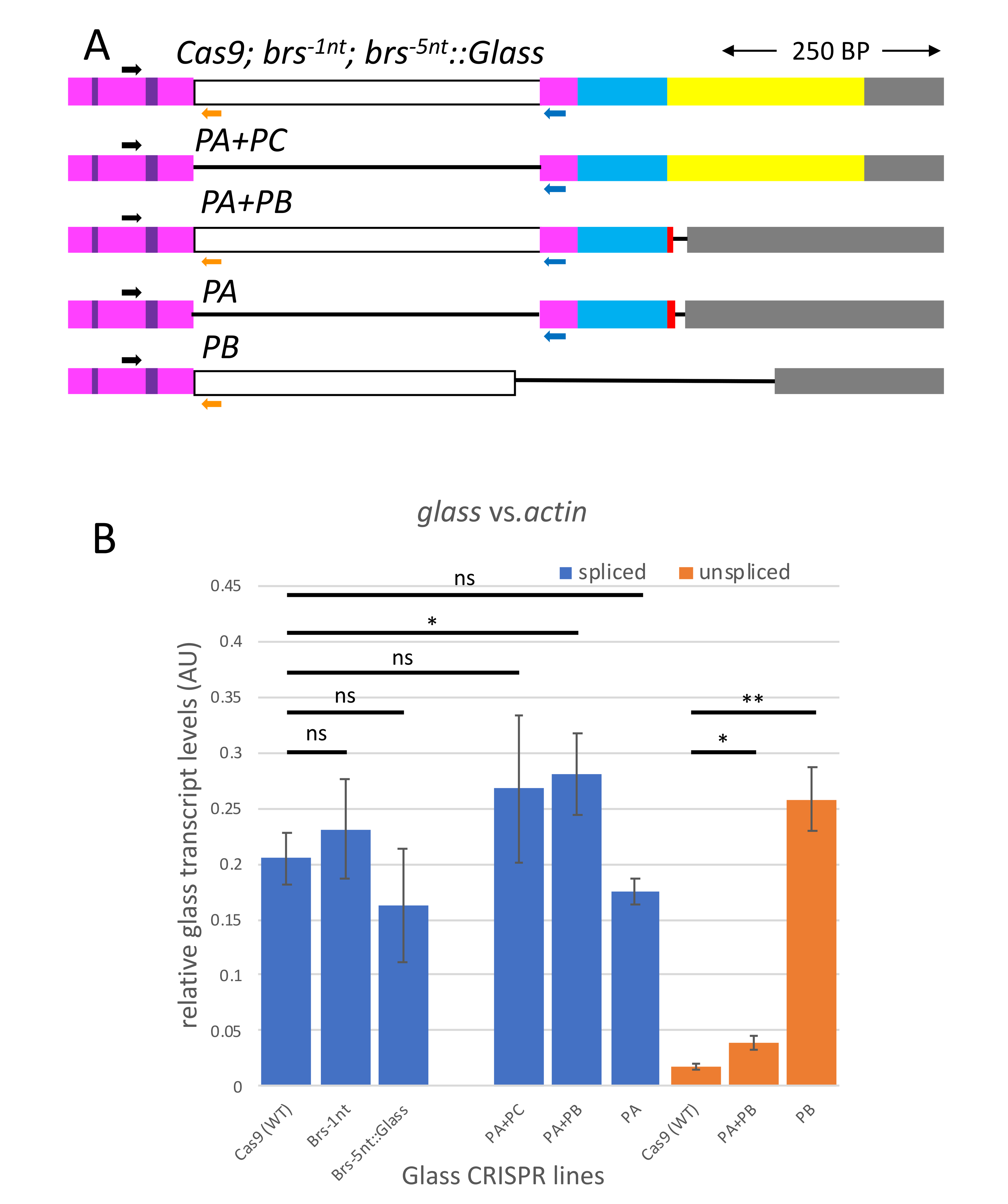

Supplement: S4 Fig — A: wildtype and mutated versions of glass from the end of exon 4 to the end of the transcript. The C2H2-zinc-finger region is shown in purple and magenta. Intron 4 is shown in white. The C-terminus of the PA isoform is shown in blue, that of the PC isoform in yellow. The 3’UTR is grey. Deletions are indicated as black lines. The triple stop codon introduced in the PA+PB and the PA alleles are indicated by red boxes. The positions of the primers used for qPCR mutagenesis are shown as arrows. Black arrow: primer “gl qP ex4 fw”; orange arrow primer “gl qP int4 re”; blue arrow: primer “gl qP ex5 re”. B: Relative amounts of spliced (blue) and unspliced (orange) glass transcripts in different CRISPR generated lines. Paired samples two-tailed t test: For all data sets n = 3 experiments. brs-1nt: p = 0.268; brs-5nt::Glass: p = 0.193; PA+PC spliced: p = 0.345; PA+PB spliced: p = 0.026; PA spliced: p = 0.265; PA+PB unspliced: p = 0.046; PB unspliced: p = 0.004. Data show mean and error bars show standard deviation. ns = not significant; * = p<0.05; ** = p<0.01. (TIF) [file pgen.1008269.s004.tif]

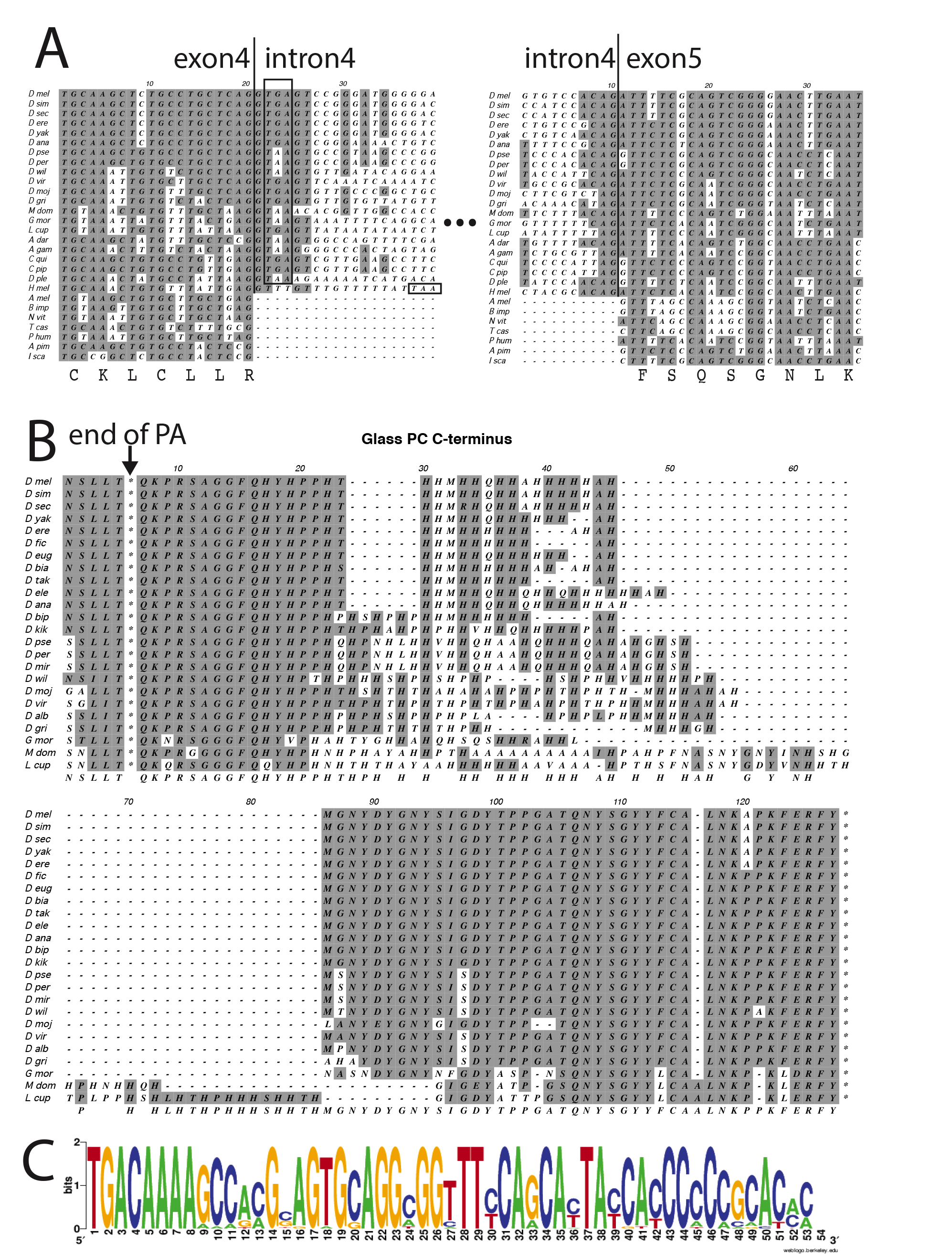

Supplement: S5 Fig — A: nucleotide sequence of the transition between exon 4 and intron 4 and between intron 4 and exon 5 of Drosophila melanogaster. Other Diptera and Lepidoptera also contain this intron followed by a stop codon black box) immediately after the exon intron junction (black line), except in Heliconius, where the stop codon is located 17 pb downstream of the exon intron junction. Although the intron is absent in other insect species, the amino acid sequence flanking the intron and forming part of the Glass zinc-finger is highly conserved as indicated by the translation below the alignment. B: amino acid alignment of the Glass C-termini of different higher Diptera. The position of the Glass PA stop codon is marked by an asterisk (arrow). The amino acid sequence directly following the end of the PA isoform is highly conserved. There is also high sequence conservation at the C-terminus of the PC isoform. The central region, which is rich in histidine residues, is more variable. Drosophila melanogaster (D mel), Drosophila simulans (D sim), Drosophila sechellia (D sec), Drosophila erecta (D ere), Drosophila yakuba (D yak), Drosophila ananassae (D ana), Drosophila pseudoobscura (D pse), Drosophila persimilis (D per), Drosophila willistoni (D wil), Drosophila virilis (D vir), Drosophila mojavensis (D moj), Drosophila grimshawi (D gri), Musca domestica (M dom), Glossina morsitans (G mor), Lucilia cuprina (L cup), Anopheles darlingi (A dar), Anopheles gambiae (A gam), Culex quinquefasciatus (C qui), Culex pipiens (C pip), Danaus plexipus (D ple), Heliconius melpomene (H mel), Apis melifera (A mel), Bombus impatiens (B imp), Nasonia vitripennis (N vit), Tribolium castaneum (T cas), Pediculus humanus (P hum), Acyrthosiphon pisum (A pim), Ixodes scapularis (I sca), Drosophila ficusphila (D fic), Drosophila eugracilis (D eug), Drosophila biarmipes (D bia), Drosophila takahashii (D tak), Drosophila elegans (D ele), Drosophila bipectinata (D bip), Drosophila kikkawai (D kik). C: WebLogo [file pgen.1008269.s005.tif]

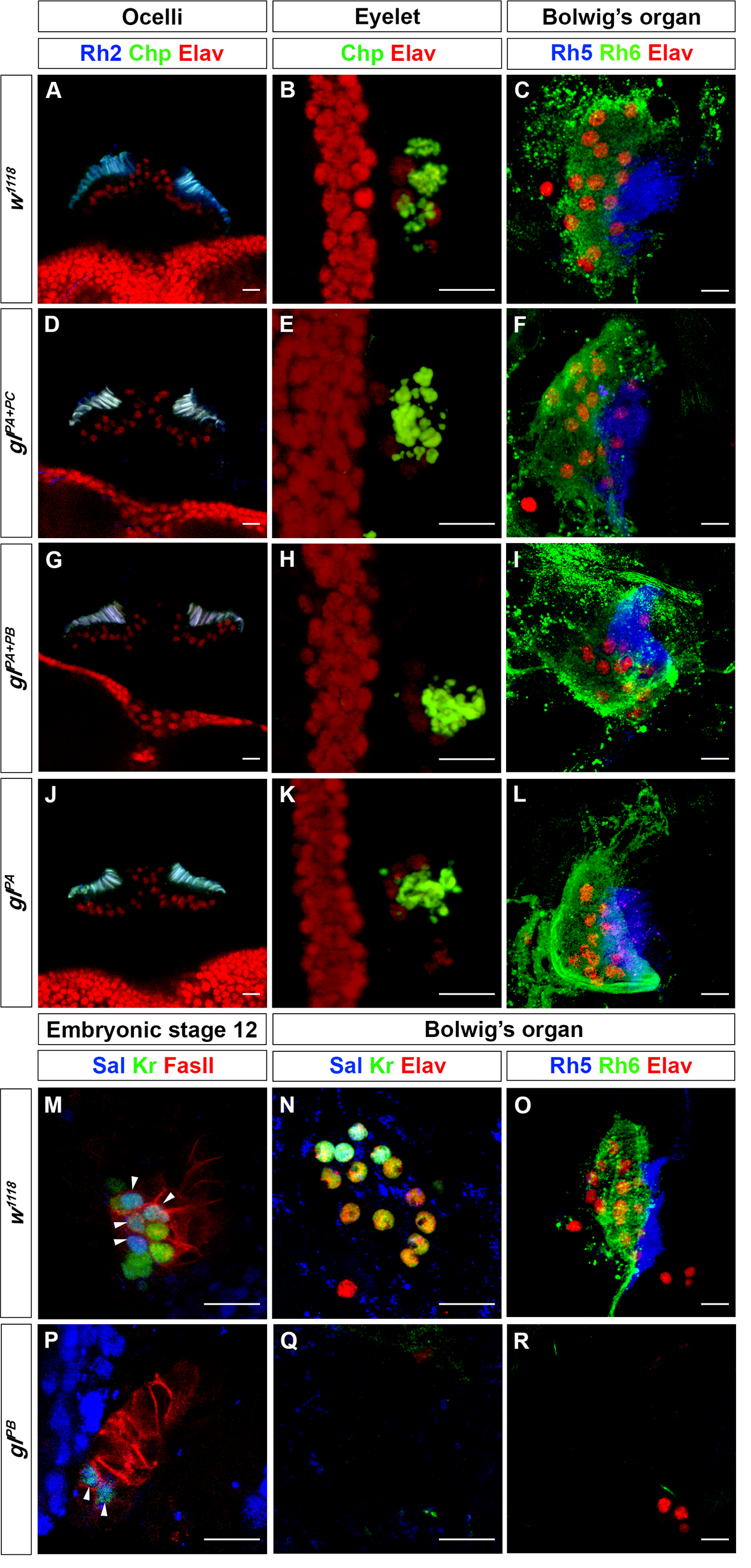

Supplement: S6 Fig — A-R: antibody staining for photoreceptor markers in ocelli, eyelet, and Bolwig’s organ of w1118 and different isoform lines as indicated on the left. Scale bars represent 10μm. A, D, G, J: ocelli stained for Rhodopsin2 (Rh2, blue), Chaoptin (Chp, green), and Elav (red) which also stains neurons of the brain located underneath the ocelli. B, E, H, K: eyelet photoreceptors stained for Chp (green) and Elav (red), which also stains neurons in the lamina (row of cells in the left part of the panels). C, F, I, L, O, R: Bolwig organ photoreceptors stained for Rhodopsin 5 (Rh5, blue), Rhodopsin 6 (Rh6, green), and Elav (red). The markers are expressed in flies expressing the Glass isoforms PA+PC, PA, and PA+PB, but not in flies expressing only the PB isoform indicating that in this case the cells are not fully differentiated or are missing completely. M, P: Bolwig organ precursors are detectable at embryonic stage 12 due to their expression of Krüppel (Kr, green), Fasciclin II (FasII, red), and Spalt (Sal, blue, arrowheads), which is only expressed in the four primary photoreceptor precursors giving rise to the Rh5 expressing photoreceptors. The precursor cells are present in embryos expressing only the Glass PB isoform, but only two of them express the markers Kr and Sal (arrowheads). N, Q: Bolwig organs stained for Sal (blue), Kr (green) and Elav (red). In wildtype Kr and Elav are expressed in all photoreceptors, while Sal is a marker for the four photoreceptors expressing Rh5. In larvae that have only the Glass PB isoform, none of the markers are expressed. (TIF) [file pgen.1008269.s006.tif]

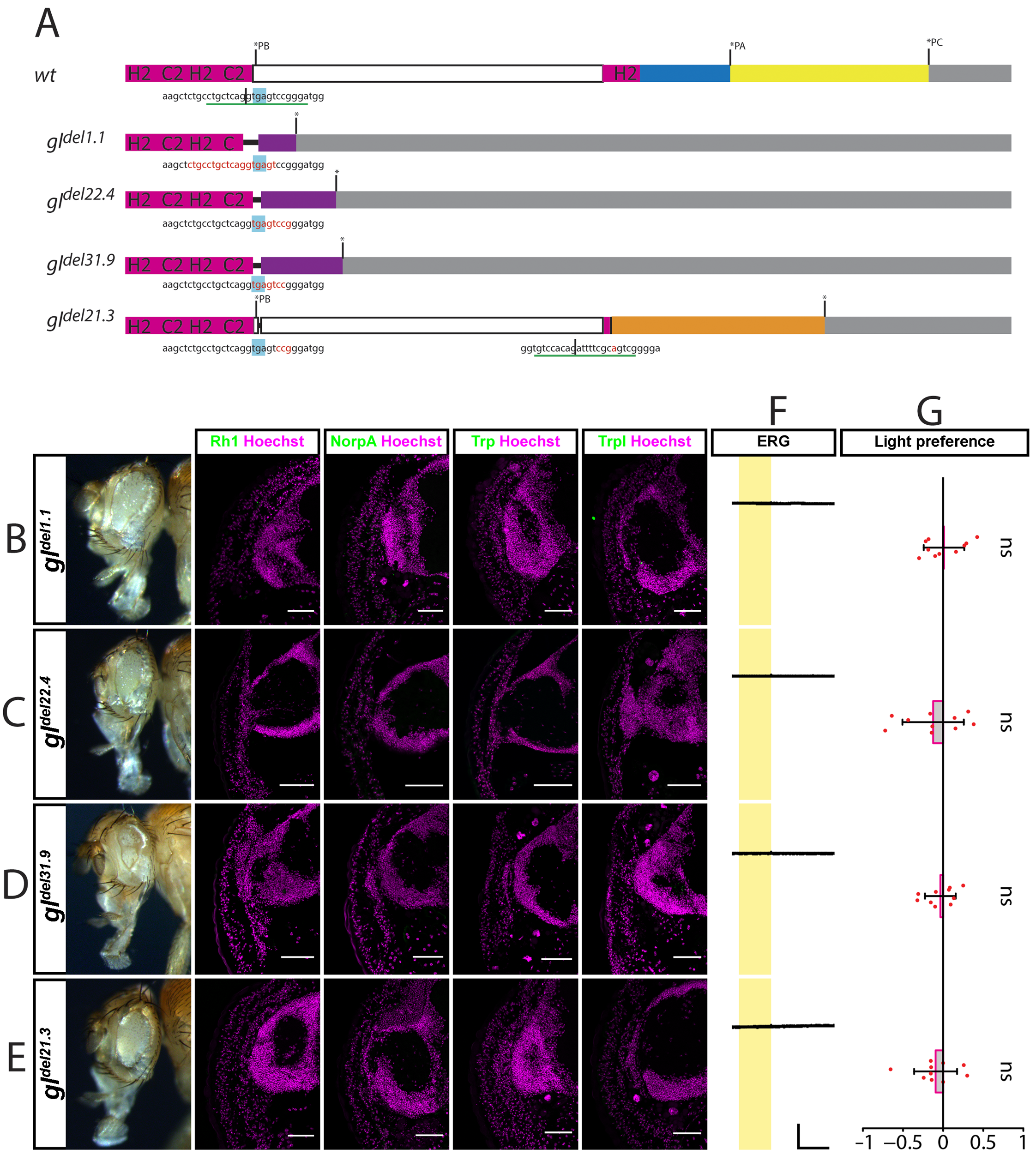

Supplement: S7 Fig — A: wildtype and mutated versions of glass from the end of exon 4 to the end of the transcript. Stop codons of isoforms PA, PB, and PC are indicated by asterisks. The C2H2-zinc-finger region is shown in magenta. Intron 4 is shown in white. The C-terminus of the PA isoform is shown in blue, that of the PC isoform in yellow. The 3’UTR is grey. The sequences at the exon intron and intron exon junctions are given as letters with a black vertical line depicting the position of the junction. The stop codon at the beginning of exon 4 is highlighted in blue. The positions of the CRISPR sites used for mutagenesis are underlined in green. Deletions are indicated as black lines in the schemes, and as red letters in the sequences. Due to the deletions of the exon intron junction and the stop codons, additional amino acids are added to the Glass PB sequence until they reach the next stop codon in intron 4 (purple boxes). Due to the frameshift caused by the single nucleotide deletion in exon 5, the amino acid sequence of the Glass PA isoform gets shifted in gldel21.3 (orange box). B-E: Adult eye phenotype and expression of the retinal markers Rh1, NorpA, Trp, and Trpl of the gldel1.1 mutant (B), of the gldel22.4 mutant (C), of the gldel31.9 mutant (D), of the gldel21.3 mutant (E). All antibody stainings are shown in green, counterstaining of DNA with Hoechst (magenta). None of the tested photoreceptor makers is expressed in these glass alleles. Scale bars: 40 μm F: ERGs of the different deletion alleles show no response to light; scale bars represent 5 mV (vertical) and 5 seconds (horizontal). G: Flies homozygous for the different small deletions in the glass locus are photoneutral. Two-tailed one sample t test followed by the Benjamini Hochberg procedure: For all data sets n = 10 experiments. gldel1.1: p = 0.90, t(9) = 0.13; gldel21.3: p = 0.53, t(9) = -1.11; gldel22.4: p = 0.49, t(9) = -1.03; gldel31.9: p = 0.66, t(9) = -0.57. The light preference index of all experimental group [file pgen.1008269.s007.tif]
